# What Is the Current Status of Research on the Impact of Digitalization in Medicine? A Bibliometric Analysis

**DOI:** 10.3390/healthcare13020093

**Published:** 2025-01-07

**Authors:** Gabriela Badareu, Silviu Cârstina, Felicia Militaru, Marian Ilie Siminică, Daniel Cîrciumaru

**Affiliations:** 1 Department of Finance, Banking and Economic Analysis, Faculty of Economics and Business Administration, University of Craiova, 200585 Craiova, Romania; silviu.carstina@yahoo.com (S.C.); marian.siminica@edu.ucv.ro (M.I.S.); danielcirciumaru@yahoo.com (D.C.); 2 Department of Psychiatry, University of Medicine and Pharmacy of Craiova, 200349 Craiova, Romania; feliciobanu@yahoo.com

**Keywords:** digitalization, medicine, trends, medical research, bibliometric analysis, VOSviewer

## Abstract

**Objectives:** This study conducts a bibliometric analysis to map key trends in the digitalization of medicine, a rapidly evolving field that incorporates advancements such as artificial intelligence, telemedicine, and Big Data. The analysis aims to identify the most prolific authors, highly cited works, leading countries, and contributions from research institutions, while also exploring emerging trends through keyword analysis. **Methods:** A dataset comprising 2606 scientific papers was retrieved from the Web of Science database. The analysis was performed using bibliometric techniques and VOSviewer software to evaluate citation patterns, author productivity, country activity, and institutional contributions. **Results:** The findings reveal a substantial increase in research activity on the digitalization of medicine, with notable contributions from Germany, the USA, and China. These countries host leading academic institutions actively driving the field. Keyword analysis highlights emerging trends in artificial intelligence, telemedicine, and Big Data. Extensive international collaborations further underscore the global nature of this research domain. This study provides a comprehensive overview of the evolution and current trends in the digitalization of medicine. **Conclusions:** It highlights the significant role of international collaboration and identifies key areas of development, offering valuable insights for future research directions in this dynamic sector.

## 1. Introduction

Digitalization in medicine represents a fundamental transformation of healthcare systems, significantly impacting how medical services are delivered, patient data are managed, and new technologies are implemented. According to Overkamp [1], digitalization in medicine is viewed by some as a revolution, while others remain skeptical about its benefits, as digital tools are often unfamiliar to healthcare professionals and their benefits are not always clear. Digitalization has become an emerging trend in healthcare, offering significant potential and generating both substantial opportunities and considerable challenges [2,3].

In the current context, the digitalization of the healthcare sector has accelerated the development process [4]. Furthermore, digitalization reduces informational asymmetries, enhancing economic efficiency and transforming various sectors, with this positive impact extending to the medical and healthcare markets [5]. This transformation includes the use of information technologies, the internet, and other digital solutions to improve accessibility, efficiency, and the quality of medical care [6]. In recent decades, digitalization has been identified as a key factor in the modernization of healthcare systems, promoting real-time access to information and facilitating communication between healthcare professionals and patients [7].

One of the most important aspects of digitalization in medicine is the integration of artificial intelligence (AI) and Big Data into medical decision-making processes. The use of advanced algorithms for diagnosis and personalized treatments can significantly improve the accuracy and efficiency of medical interventions [8]. Additionally, telemedicine, which has rapidly expanded in recent years, facilitates remote consultations, thereby reducing geographical and economic barriers while providing patients with real-time access to healthcare [9]. Moreover, digitalization plays a crucial role in developing healthcare infrastructure, including the implementation of electronic health records (EHR), which ensures rapid and secure access to patients’ medical histories. These technologies enable better data management, reduce errors, and improve the quality of care [10].

At the same time, it contributes to improving the efficiency of healthcare systems by automating administrative and medical processes and promoting a more financially sustainable care model [11,12]. However, the challenge to greater efficiency in digital medicine lies in the rapid pace of technological progress, which exceeds the current capacity for practical implementation. The generations involved have different understandings of technology, and there is a lack of curriculum training in medical schools. Therefore, a significant improvement in digital medical skills training is needed so that current and future healthcare professionals are better prepared for digitalized medicine [13].

Consequently, digitalization in medicine is not only revolutionizing how medical services are provided but also radically changing the paradigm in which health information is managed and processed, facilitating the creation of more efficient and accessible healthcare systems worldwide [14,15].

Digitalization in medicine is a continuously expanding field that significantly impacts healthcare systems worldwide. The implementation of digital technologies in healthcare includes a wide range of applications, including telemedicine, artificial intelligence (AI), Big Data, computerized medical record management systems, and interoperability. These technologies have introduced essential innovations that significantly impact the efficiency, accessibility, and quality of medical care. In recent decades, digitalization has been recognized not only as a necessity but also as an opportunity for the profound transformation of healthcare services [16,17]. Based on the literature, we define digitalization in medicine as the process of integrating and utilizing advanced digital technologies—such as telemedicine, artificial intelligence (AI), Big Data, electronic medical records management, and interoperability systems—to transform and improve the delivery of healthcare services. This definition highlights the role of digitalization in enhancing efficiency, accessibility, and quality of medical care while revolutionizing the management and processing of health information.

Bibliometric analysis in the field of digitalization in medicine has become an essential method for understanding research trends and the impact of digital technologies on healthcare. These studies provide a clear view of the evolution of research in this field, identifying influential authors, key institutions, and emerging trends in the scientific literature [18].

In the specialized literature on digitalization in medicine, bibliometric analyses are becoming increasingly common due to the advantages of this technique. However, no bibliometric analyses have been found that investigate digitalization across the entire field of medicine. Instead, most studies focus on specific subfields, such as cardiology [19], mental health [20,21,22], and other specialties [23,24,25,26]. This suggests a lack of comprehensive bibliometric approaches that examine the impact of digitalization on the entire medical sector, thus presenting an unexplored research opportunity in this context.

To better understand the evolution of this field and identify future research directions, the aim of this paper is to conduct a detailed bibliometric analysis of the literature on digitalization in medicine. This analysis will focus on identifying the most prolific authors, research institutions, and keywords, as well as evaluating publication trends in the field. The paper aims to synthesize research in this area and contribute to a deeper understanding of the digital progress and challenges in healthcare, thereby helping to formulate clear directions for future studies. This study addresses the existing gap by conducting a bibliometric analysis that examines the impact of digitalization in medicine across all medical subfields, rather than limiting the scope to individual specialties. In doing so, we provide a broader, more holistic view of how digital technologies are transforming healthcare as a whole. This approach offers a deeper understanding of overall trends, emerging research hotspots, and the interconnectedness of digitalization in various aspects of medicine, which is currently underexplored in existing studies.

Thus, this study contributes to the literature by providing a comprehensive analysis of digitalization in medicine. It helps synthesize research across multiple disciplines and provides valuable insights for future studies and the continued development of digital health.

This analysis will address the following research questions:

- RQ1: What is the trajectory of academic articles published on digitalization in medicine?
- RQ2: Who are the most prolific authors in this field?
- RQ3: Who are the most co-cited authors in studies on digitalization in medicine?
- RQ4: Which countries have the most publications on digitalization in medicine?
- RQ5: Which research institutions have contributed significantly to digitalization studies in medicine?
- RQ6: What are the most common keywords used in the literature on digitalization in medicine?
- RQ7: What are the most cited works on digitalization in medicine?

The paper is structured as follows: Section 1 establishes the theoretical framework, while the literature review identifies gaps and trends in digitalization research within medicine. Section 2 details the methodology for the bibliometric analysis, while the Section 3 highlight the main findings. Section 4 synthesizes the major contributions and provides recommendations for future research in this field, emphasizing developmental directions and emerging challenges.

## 2. Materials and Methods

Bibliometric methods have been used to provide a quantitative analysis of written publications [27]. These methods serve as a research technique to measure and analyze the performance and structure of academic research using quantitative indicators [28]. As demonstrated by Wang et al. [29], bibliometric analysis is a valuable tool for assessing scientific performance and research trends within specific fields. This technique is essential for evaluating research trends, citation patterns, and academic collaborations, offering insights into emerging fields and identifying influential authors and institutions. In dynamic fields like digital health, bibliometric analysis plays a crucial role in mapping intellectual structures and assessing research impact [30,31,32]. Bibliometric analysis and other related methods stem from several key themes, such as co-citation mapping and co-citation link analysis [33]. Given the rapid growth of research output in areas such as digital health and healthcare technologies, bibliometric methods are increasingly being utilized to assess research impact and map the intellectual structure of these disciplines [34,35]. This type of analysis is typically based on databases that include information about research papers published in the field of interest [36]. One widely used database is Web of Science (WOS), which hosts an extensive collection of research papers. The online WOS database includes nearly all significant research papers and offers integrated analysis tools to generate representative figures [37]. Both Web of Science and Scopus are increasingly used in academic papers [38,39,40]. A comprehensive study by Liu and He [41] examined the growth and trends in scientometric studies using Web of Science Core Collection data from 1992 to 2020. They highlighted that Web of Science is one of the most widely utilized databases for scientometric research, underscoring its relevance in conducting bibliometric analysis. Also, Nicolaisen [42] outlines the history and evolution of bibliometric analysis, emphasizing Web of Science as a vital data source. Gu et al. [43] conducted a bibliometric analysis of digital health research, emphasizing the importance of the Web of Science database as the primary data source. The authors highlighted that citation patterns and co-authorship networks were analyzed using Web of Science data to uncover emerging trends and developments in the field of digital health. These findings underscore the reliability and significance of Web of Science as a valuable resource for conducting comprehensive bibliometric studies in health-related domains. Their work aligns with other studies that also rely on Web of Science for accurate and reproducible bibliometric analyses in digital health and other scientific fields, emphasizing the importance of data from Web of Science for conducting impact analysis.

In this research, we utilized Web of Science (WOS) and applied the keyword “digitalization in medicine” with the condition that it “must be included” to ensure the relevance of the selected articles. This methodology guarantees that our analysis covers a complete and representative range of data within the specified categories. Additionally, to ensure a comprehensive and representative analysis, we included all sub-categories of Web of Science (SCI-EXPANDED, SSCI, AHCI, CPCI-S, CPCI-SSH, BKCI-S, BKCI-SSH, ESCI, CCR-EXPANDED, IC) as outlined in the reference. These sub-datasets were fully applied to ensure that no relevant works were omitted. A filter was applied to include only articles where this exact phrase appeared, either in the title, abstract, or keywords. By applying this specific filter, we aimed to target publications that directly discuss the integration of digital technologies in healthcare, their applications, and their impact on medical systems. The inclusion criteria guaranteed that the term digitalization in medicine was central to the content of the articles, providing a robust foundation for our bibliometric analysis. This approach generated a dataset of 2606 published papers between 1975 and 2024. No additional filters for language, document type, research area, or any other criteria were applied in this study, as the primary objective was to ensure a comprehensive and unbiased exploration of the research landscape on the topic. By including all available document types and languages, the analysis aimed to provide a holistic perspective, capturing the full spectrum of scientific contributions without restrictions that could limit the inclusivity or breadth of the results. Consequently, the study included documents in a range of languages, as per the Web of Science database. The distribution of included documents by language is as follows: English: 2291, German: 243, Russian: 27, Spanish: 15, French: 9, Chinese: 7, Italian: 7, Bulgarian: 1, Greek: 1, Korean: 1, Polish: 1, Slovenian: 1, Turkish: 1, Ukrainian: 1. The WOS database format allows it to be used for bibliometric analysis by exporting data to specialized software, such as the VOSviewer 1.6.18 tool. VOSviewer can display three types of map visualizations: network, overlay, and density [44]. In the network visualization, each keyword, author, or country/organization is represented by a circle (node), with the size of the circle proportional to the number of publications in which the analyzed unit is mentioned [45]. Each color represents a group of analyzed units, the length of the curved lines between circles indicates the approximate connection of the analyzed units, and the thickness of these lines shows the strength of the link between thematic pairs [46,47]. Clusters represent the relationship between one subject and another [48]. This scientific software, VOSviewer, helps extract data, create maps, and group articles from datasets containing bibliographic fields (title, author, keywords, journal, affiliations, etc.) [49,50,51,52].

In our research, we analyzed citation, co-citation, occurrence, co-occurrence, and co-authorship. Citation analysis is an essential method in scientific mapping, based on the premise that citations indicate intellectual links between works when one publication refers to another [53]. Bibliometric analysis aids in author citation by providing a systematic approach to measuring and tracking scholarly impact. As demonstrated by certain authors, this type of analysis plays a crucial role in identifying the most prolific researchers in a given field [54,55,56]. Co-citation analysis, on the other hand, assumes that works frequently cited together address similar themes [57]. While citation and co-citation analyses focus on scientific papers, co-word analysis examines the words found in titles, abstracts, and full texts. Typically, the terms analyzed are extracted from the “author keywords”, and in their absence, meaningful words from other sections of the paper, such as titles or abstracts, may be used [58,59,60]. Finally, co-authorship analysis examines collaborations between researchers in a specific field. Co-authorship represents a formal form of intellectual cooperation between researchers [61], and analyzing these collaborations helps to understand how researchers share knowledge and contributions.

**Figure 1:**
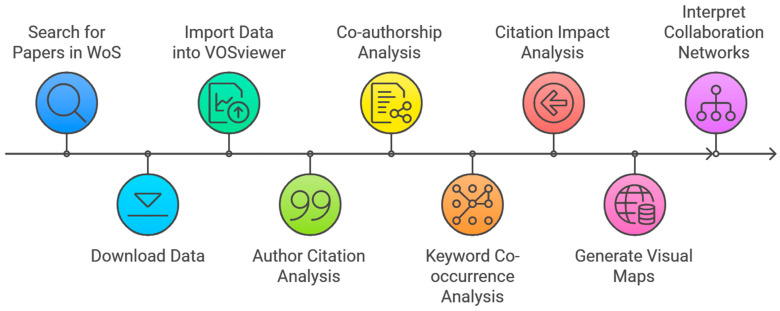
Steps of bibliometric analysis. Source: Author’s own processing.

To provide a clear understanding of the methodology applied in this research, we present a flow diagram that outlines the key stages of the analysis (see Figure 1). This workflow systematically demonstrates the steps taken to ensure a rigorous and comprehensive examination of the dataset.

## 3. Results

### 3.1. The Evolution of Scientific Publications in the Digitalization of Medicine

**Figure 2:**
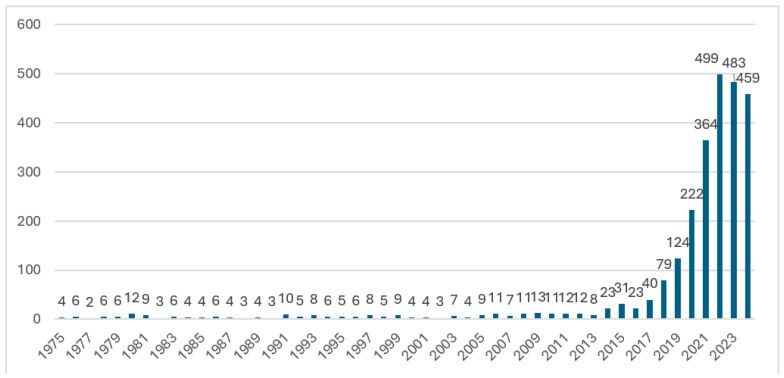
Evolution of publications over the years. Source: Author’s own elaboration using the Web of Science database.

To answer RQ1, I analyzed the evolution of publications on the topic of digitalization in medicine, reflecting the technological progress and social transformations that have influenced the adoption of digital technologies in this field. Starting from the 1970s, the number of publications remained relatively constant and low (as shown in Figure 2), indicating limited exploration of the topic during that period. The early papers laid the groundwork for fundamental concepts in medicine without directly addressing digitalization.

After the 2000s, the number of publications on this topic began to grow significantly as information technology and internet access became more widespread. During this period, digitally integrated medical devices and new technologies, such as artificial intelligence, opened up new perspectives on diagnostics and personalized treatments. The publication rate steadily increased starting in 2010, driven by the growing emphasis on digital transformation in healthcare, remote monitoring, and medical data analysis.

Thus, we can conclude that the reduced number of publications before the 1990s, as shown in Figure 2, can be attributed to a combination of factors. The limitations of the Web of Science Core Collection in retrieving older literature, as discussed in prior research, alongside the early stages of digitalization in medicine before the 1990s, both contributed to the low volume of published work during this period [31]. As Web of Science expanded its archival coverage and as digital health research gained momentum with advancements in computational methods, networking technologies, and the adoption of electronic health systems in the 1990s, research output in this field began to grow more significantly. These factors collectively provide an explanation for the trends observed in the bibliometric analysis and emphasize the importance of considering technological evolution and database limitations when interpreting publication trends in rapidly advancing fields like digital health.

The period from 2020 to 2024 stands out for its prolific output, reaching a peak in 2022 with 499 publications. This increase can be attributed to several interconnected factors. First, the COVID-19 pandemic acted as a major catalyst for digitalization in medicine. Mobility restrictions and the need for social distancing accelerated the implementation and research of digital solutions, such as telemedicine, remote monitoring, and mobile health applications. Publications during this period predominantly focused on using these technologies to manage the health crisis and improve access to healthcare.

Second, technological progress has significantly contributed to this trend. The development of artificial intelligence, Big Data, and other technological innovations has enabled the rapid analysis of large volumes of clinical data, leading to more accurate diagnoses and personalized treatments. In 2022 and 2023, there was increasing interest in integrating robotics and automated solutions into digital medicine.

Additionally, global interest in digital health has been driven by challenges such as an aging population and the rising incidence of chronic diseases. These trends emphasized the need for effective digital solutions to support diagnostics, prevention, and patient-tailored treatments.

In addition, the abnormal increase in publications observed in the dataset may be attributed to the sudden inclusion of a new set of data, as highlighted in the study by Liu [62]. Another possible factor explaining the significant increase in publications is the expansion of the Web of Science Core Collection, particularly in recent years. The Web of Science Core Collection, one of the most authoritative bibliographic databases, is widely used in academia to track high-quality research [63] According to studies documented in Scientometrics [64,65,66], as the database has incorporated more diverse and recent sources, an increase in the volume of available publications has been observed. This suggests that the expansion of the database may significantly contribute to the growth in the number of publications. Therefore, this factor should be considered an additional cause of the trends observed in our bibliometric analysis.

In response to RQ1, the recent peak in publications in the field of digitalization in medicine reflects the scientific community’s adaptation to significant global events, such as the COVID-19 pandemic, as well as the new opportunities created by technological advancements. However, it is important to emphasize that earlier works played a fundamental role in this process by providing a solid conceptual foundation for subsequent research. These initial studies demonstrate that digitalization in medicine is not a spontaneous phenomenon; rather, it is the result of a gradual evolution that combines scientific tradition with technological innovations.

As the number of publications evolved, so did the concept of digitalization in medicine. As a result, before 2000, the literature primarily focused on conceptual introductions and theories regarding the use of information technologies, but without direct applicability in clinical practice. During this early period, emphasis was placed on the potential of technologies for data management and facilitating access to healthcare. However, the real transformation began in the period from 2000 to 2010, when digital technologies started to be integrated into healthcare systems, and the concept of “digital medicine” evolved beyond simple patient data management to include the use of these technologies for diagnosis and treatment through telemedicine platforms and electronic health records. The literature also began to reflect this shift, with an increasing number of references to the practical applications of digital tools in healthcare. 

Later, starting in 2010, the rapid development of emerging technologies such as artificial intelligence (AI), the Internet of Things (IoT), and Big Data analytics transformed digital medicine. These technologies not only expanded the use of digitalization in preventive medicine and diagnostics but also opened new possibilities for personalized treatments and remote interventions. The term “digitalization in medicine” gained widespread usage during this period, aligning with the growing body of literature exploring the integration of digital tools across medical practice. 

As the number of publications increased over time, the concept of digitalization in medicine also evolved, reflecting broader technological advancements and the expanding role of digital tools in healthcare. Today, the concept of “digital medicine” is deeply integrated into medical practice, encompassing not only telemedicine and remote diagnostics but also advanced patient monitoring solutions in real time, personalized treatments based on genetic data, and the use of AI for forecasts and personalized therapies, thus contributing to the improvement of healthcare quality and accessibility.

### 3.2. The Most Active Authors in the Field of Medical Digitalization

Analyzing the authors with the most publications in a scientific field is essential for understanding the evolution and impact of research in a particular area of study. In the case of medical digitalization, identifying the most prolific authors reflects not only scientific activity and foundational research but also innovative contributions that can shape the future of the field. Furthermore, analyzing these authors allows for a more detailed evaluation of their impact, in terms of both the number of publications and the citations received, which signify the recognition and applicability of their research within the scientific community.

In this context, analyzing the most prolific authors in the field of medical digitalization serves as a valuable tool for understanding how this topic has developed and how their research has influenced progress in the medical field. Therefore, identifying and examining these authors contributes not only to appreciating scientific progress but also to guiding new researchers and practitioners in the field of medical digitalization.

This section analyzes the authors with the most publications in the field of medical digitalization. To obtain a clear and easily understandable picture, a threshold of at least five publications per author was applied, classifying only those who met this condition as “prolific”. Out of a total of 13,329 identified authors, 111 were included in this category and were subsequently analyzed and mapped according to the number of publications and their average publication year.

The temporal distribution of publications is illustrated by the colors of the circles, ranging from dark blue, indicating works published from 1975 onwards (the first record in the Web of Science database), to bright yellow, representing recent works from 2024. The analysis reveals that only one author published more than five works in this field before 2000, represented by a turquoise circle, specifically in 1996. The remaining prolific authors primarily conducted their scientific activity after 2015.

**Figure 3:**
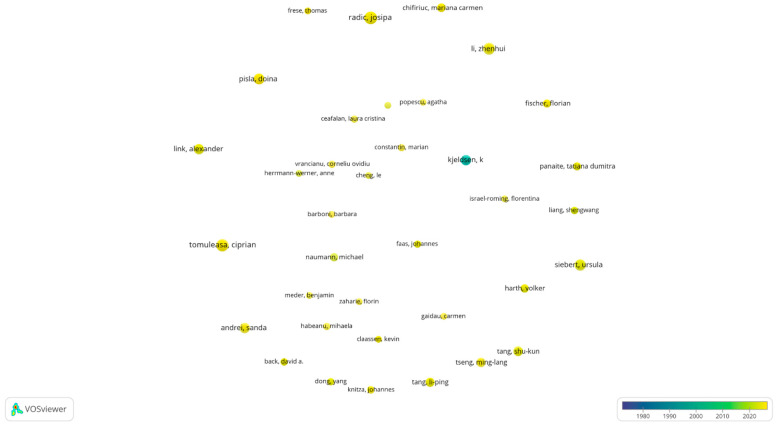
The most prolific authors. Own processing in VosViewer.

According to the Web of Science database, the most publications in the field of medical digitalization was authored by Tomuleasa C., who has published 17 scientific works. In the figure below (Figure 3), the density circle assigned to this author is yellow, indicating that his publications were produced in the last 10 years, with the average year of publication being 2023. This suggests that Tomuleasa C. is a relatively new researcher in the field but has made a significant impact with an impressive number of recent publications.

The second position in the ranking of the most prolific authors is shared by Radic J., Radic M., and Vuckovic M., each with 16 publications. The relationship between the number of works and citations suggests that these authors have frequently collaborated, resulting in identical statistics. Similar to Tomuleasa C., these researchers were assigned the yellow circle, indicating that their research was conducted recently, with the majority of their publications recorded in 2023.

The ranking continues with Li Z., who is in third place with 15 publications to date, most of which were published in 2022. Siebert U. ranks fourth with 14 works and an average publication year of 2021. In fifth place are Gelemanovic A. and Pisla A., with 12 publications each and their most recent works published in 2024.

Other notable authors include Kolak E., Kjeldsen K., Ghiaur G., Link A., Al Hajjar N., and Vaida C., who have each published 11 works. Kjeldsen K., in particular, is considered one of the pioneers in this field, having made significant contributions since 1977, which underscores his importance in the early development of research in medical digitalization. His early contributions were essential in establishing the theoretical and practical foundations for subsequent research, and his impact in the field remains significant, even though he is not among the most prolific authors in recent years.

Although authors with fewer than 10 works were not analyzed in detail, their contributions should not be overlooked. The analysis suggests that a large number of publications does not automatically guarantee a high number of citations, and their importance in the field can be significant even with a lower impact in terms of citations. This demonstrates that although the number of publications may be an indicator of scientific activity, the success and relevance of a paper are also determined by other factors, such as its applicability and the innovation it brings to the field.

In conclusion, the analysis of the most prolific authors in the field of medical digitalization provides a detailed view of the evolution of research in this field and addresses the second research question regarding the most prolific authors. Notable among these are Tomuleasa C., Radic J., Radic M., Vuckovic M., Li Z., Siebert U., Gelemanovic A., and Pisla A. Most of these prolific authors have been active in recent years, especially in the last 5–10 years, which reflects the interest and need for healthcare systems to adapt to new technologies. Furthermore, the significant contributions of some older researchers, such as Kjeldsen K., highlight the importance of foundational research and its impact on the subsequent development of this field.

### 3.3. Co-Citation Analysis of Authors in the Context of Digitalization in Medicine

**Figure 4:**
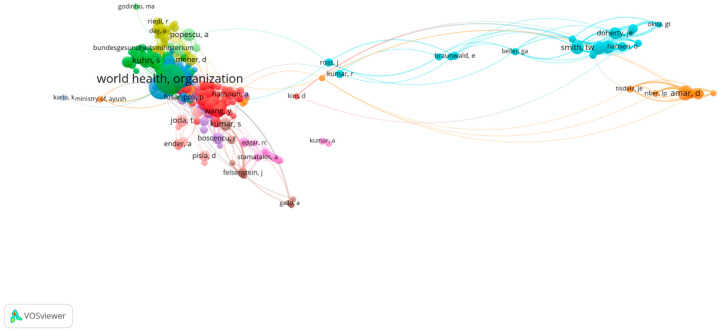
The most co-cited authors. Author’s own processing using VosViewer.

The bibliometric analysis carried out using the VOSViewer application allows for the identification of the most cited authors in a specific research field, considering both direct citations and co-citations across various publications [67]. This analysis can be used to reveal the intellectual structure of a research field [68], including its underlying themes [69]. To highlight the most influential authors in the field of digitalization in medicine, we applied a threshold of at least 10 co-citations per author. This filter enabled the identification of 392 authors with significant impact, grouped into 12 clusters, each representing collaboration networks with between 1 and 120 members, as shown in Figure 4. These clusters reflect the influence of authors on research directions and their contributions to the field.

At the top of this ranking is the World Health Organization (WHO), with an impressive 230 co-citations. The WHO is globally recognized for its authority in setting health standards and norms. Its publications are frequently cited due to their global relevance and scientific authority, playing an essential role in shaping digital health policies, as reflected in documents such as the Global Strategy on Digital Health 2020–2025, which has significantly influenced international research and policies in the field. Although the WHO is an organization rather than an individual author, its contributions remain highly relevant, as its research and documents are fundamental sources of information and recommendations in digital health. The high citation frequency of the WHO underscores not only the authority of this organization but also its global impact in promoting digitalization in medicine.

Another important actor is the European Commission, which has 124 co-citations. The Commission plays a crucial role in the development and implementation of European policies related to the digitalization of health through initiatives such as the Digital Single Market Strategy and the Digital Health Action Plan. These documents are fundamental for research in the field, providing essential data and analyses that support the development of digital solutions in health systems across the European Union. Although the European Commission is an institution and not an individual author, it is cited as a key source of documentation due to the important documents and information it provides. As a result, it is considered a significant reference source in the field of digital health.

In third place is Amar, D., with 60 co-citations, who is recognized as a reference author in the field of digitalization in medicine. His research themes include telemedicine, the use of artificial intelligence in diagnostics and personalized treatments, and the use of Big Data in medical decision-making. His research significantly contributes to the understanding of the impact of digitalization on health systems, and the practical applicability of his findings accounts for the high number of citations.

Fourth place is held by Kuhn, S., with 53 co-citations. Kuhn has explored 3D applications in creating personalized implants and planning complex surgeries, as well as the integration of artificial intelligence for personalized treatments. The broad impact of his research on improving access to and efficiency in healthcare has contributed to the frequent citation of his works.

Following in the ranking is Greenhalgh, T., with 51 co-citations, due to her significant contributions to the development of digital innovation implementation models and the assessment of digital maturity in hospitals. Her research is essential for understanding technological transformations in healthcare and their impact on the efficiency and accessibility of healthcare services.

Other notable authors include Smith, T. W. (44 co-citations), who is known for promoting the use of digital technologies to make health systems more sustainable, as well as Popescu, A. (43 co-citations) and Kumar, S. (42 co-citations), who have researched innovative solutions for managing public health through digital technologies, including electronic monitoring systems and artificial intelligence.

Additionally, organizations like the OECD, which has 41 co-citations, have had a significant impact on the standardization of digital health policies, while authors like Wang, Y. and Zhang, Y. (39 co-citations) have investigated the use of artificial intelligence and Big Data in personalizing medical treatments.

The significant number of co-citations for these authors reflects the profound impact of their works on the development of digital health. Their contributions have been essential in defining and implementing innovative technological solutions, promoting paradigm shifts in healthcare delivery. In conclusion, the co-citation analysis and the collaborations among relevant authors in the field of digitalization in medicine address RQ3 and highlight their significant impact on the development and implementation of technological solutions in health. International organizations such as the WHO and the European Commission, as well as individual researchers, have significantly contributed to advancing policies and research in this field, obtaining the highest number of co-citations. This large number of co-citations reflects not only their authority but also the relevance of their works in improving the efficiency and accessibility of healthcare services while promoting innovative digital solutions. These contributions are essential in strengthening the foundation for the digital transformation of health systems, paving the way for new directions in research and development in the future.

In conclusion, the co-citation analysis and collaborations among relevant authors in the field of digitalization in medicine address RQ3 and highlight their significant impact on the development and implementation of technological solutions in healthcare. International organizations, such as the WHO and the European Commission, as well as individual researchers, have significantly contributed to advancing policies and research in the field, obtaining the highest number of co-citations. This large number of co-citations reflects not only their authority but also the relevance of their work in improving the efficiency and accessibility of healthcare services and promoting innovative digital solutions. These contributions are essential for strengthening the foundation of the digital transformation of health systems, paving the way for new directions in research and development in the future.

### 3.4. Analysis of Countries with the Highest Number of Publications in the Field of Digitalization in Medicine

Digitalization in medicine represents one of the most important trends in the global transformation of healthcare systems, as it is essential for improving access to medical services, reducing costs, and increasing the efficiency of their delivery. In this context, analyzing scientific publications by country serves as a valuable method for understanding how different nations contribute to this emerging field. At the conclusion of the analysis, we will be able to answer RQ4.

This analysis provides insights into the geographic distribution of research in digital health, revealing national investments and priorities. The number of publications reflects the level of involvement and resources allocated by each country to promote digital health.

**Figure 5:**
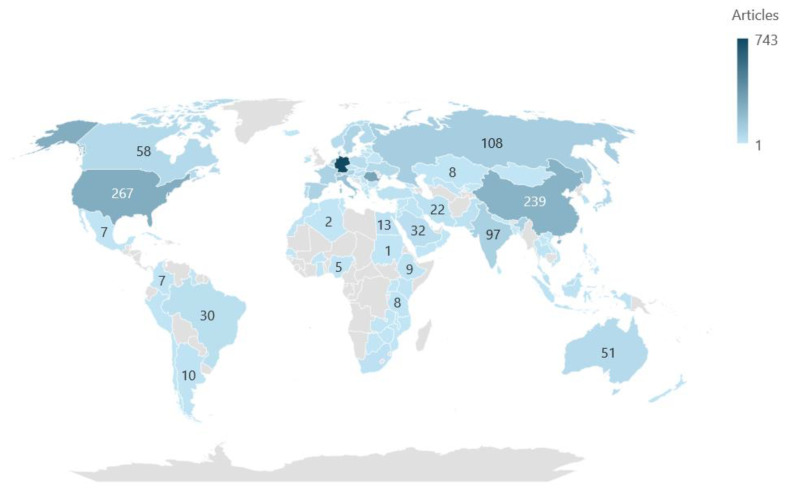
The most prolific countries. Source: Authors’ own processing using VosViewer.

According to the Web of Science database and the map in Figure 5, Germany leads in the number of publications (743 publications) in the field of digitalization in medicine. The country is recognized for its significant investments in research and innovation, particularly in digital health. Additionally, it benefits from a well-developed healthcare system, world-renowned universities, and strong international collaborations. Public policies also support digitalization through initiatives such as Digital Health Germany.

Following Germany is Romania (308 publications), which stands out with its high position. The large number of publications can be attributed to the increased academic interest in the digitalization of Romanian medicine, aiming to integrate digital solutions to address accessibility and efficiency issues within the healthcare system. Moreover, support for research and development in this field has grown in the context of adopting the European digital agenda.

In third place is the United States, with 267 articles. Although the U.S. is a leader in many research fields, its lower position in this ranking may be due to specific categorization or incomplete databases. However, American universities and medical technology companies (such as Microsoft and Google Health) contribute significantly to innovation. Following in the ranking is China, with 239 articles. Substantial investments in artificial intelligence and digital health technologies have enabled China to become an important global player. The government promotes digital transformation initiatives in hospitals and medical supply chains, which accounts for the large number of publications.

Italy (168 publications) and the United Kingdom (161 publications) rank fifth and sixth, respectively. These countries stand out for their strong commitment to digital health, evidenced by well-defined national strategies and the implementation of clear policies in this field. In Italy, initiatives such as the integration of electronic health records and the development of telemedicine infrastructure have contributed to its leadership in scientific production. Similarly, the UK has been a pioneer in the use of digital technologies in healthcare through initiatives like NHS Digital, which supports both research and the large-scale adoption of digital solutions. Both countries benefit from international collaborations and European funding programs, such as Horizon Europe, which facilitate the development of relevant and innovative studies.

Switzerland (129 publications) and the Netherlands (127 publications), although smaller in terms of population, have shown remarkable performance in digital health. This success is due to their top-tier, well-organized medical systems, which support innovation and research. In Switzerland, the combination of advanced medical infrastructure and a solid academic culture contributes to the production of high-quality scientific papers. The Netherlands, on the other hand, is recognized for its patient-centered digital health initiatives, including the development of telemonitoring applications and the use of artificial intelligence for diagnostics.

Among the top 10 most prolific countries is Russia, with 106 publications. Although it is active in digital health research, Russia faces some limitations that influence its total number of publications. These barriers include insufficient funding and restricted access to international collaborations, which reduce opportunities to participate in joint research projects with other countries. However, Russia continues to develop programs and initiatives in digital health, indicating a sustained interest in this field. In tenth place is Austria (102 publications), which demonstrates a solid commitment to integrating digital solutions into its healthcare system. This success is facilitated by a well-developed medical infrastructure that supports the use of advanced technologies, such as electronic records and telemedicine. Furthermore, Austria’s participation in European initiatives and collaborations with other EU countries significantly contributes to its academic output. Cross-border collaborations provide access to additional resources and foster a continuous exchange of knowledge and best practices in digital health.

Countries that follow the top ten in publication rankings, such as India (97 publications), Spain (89 publications), France (88 publications), Sweden (85 publications), Denmark (63 publications), and Japan (61 publications), also demonstrate a significant interest in advancing digital health. India, for example, focuses its efforts on digital solutions aimed at enhancing accessibility and inclusion, given the challenges posed by a healthcare system that must serve a large and diverse population. Spain and France, as beneficiaries of European initiatives, contribute through the development of interdisciplinary projects that combine medicine with emerging technologies, such as artificial intelligence and the Internet of Things (IoT). Sweden and Denmark, known for their well-organized healthcare systems, invest in applied research to improve digital healthcare services, with a focus on personalized solutions for patients. Japan, in turn, remains a global technological leader, contributing to digital health through innovations in medical robotics and telemedicine. Each of these countries makes essential and specific contributions, reflecting their national interests and local challenges, while demonstrating a global commitment to the development of digital health.

In conclusion, the analysis of publications by country in digital health highlights a global distribution of interest and contributions, reflecting national priorities, technological capabilities, and local challenges. Leading countries like Germany, Romania, the United States, and China drive research due to their financial and academic resources, advanced digital infrastructure, and international collaborations. At the same time, smaller countries such as Switzerland, the Netherlands, and Austria demonstrate that academic excellence and well-defined strategies can compensate for the lack of extensive resources. Contributions from other nations, including India, Japan, Spain, and France, emphasize the diversity of global initiatives, ranging from innovations in robotics and artificial intelligence to accessibility in medical services for vulnerable populations. Overall, this analysis underscores the importance of digital health as an international research and development priority, showing how different approaches and global collaborations can lead to significant advances in improving healthcare services and strengthening public health in the digital age.

### 3.5. Co-Authorship by Organizations

In the previous analysis, we examined the impact of digitalization in medicine at the national level, highlighting the key countries involved in this field. This approach provided a general overview of the regions actively investing in research and development in medical digitalization. However, to gain a deeper understanding of how research is conducted in this sector and the concrete contributions being made, it is essential to expand the analysis to include academic and research institutions.

Thus, in this context, we aim to move beyond a country-based ranking and focus on the top universities and institutions that play a key role in the digitalization of medicine. This approach will allow us to identify centers of excellence in the field, gain a better understanding of the structure and international collaborations that support technological development, and observe how different institutions contribute to the progress of this sector, even in countries with more limited resources compared to major research powers.

**Figure 6:**
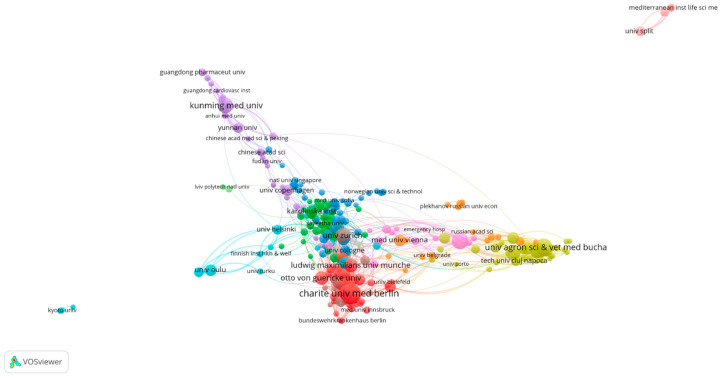
Co-authorship among institutions. Source: Author’s own processing using VosViewer.

According to Figure 6, in the field of medical digitalization, several higher education institutions have made significant contributions through scientific publications and international collaborations. Among these, Charité University Medicine Berlin stands out with an impressive total of 76 published articles and 64 collaborations, both national (e.g., Bundeswehrkrankenhaus Berlin, Ludwig Maximilian University of Munich) and international (e.g., University of Zurich, Karolinska Institute), reflecting a vast network of international partnerships and significant influence in research in this field. Charité University Medicine Berlin ranks first due to its extensive research networks and global impact.

Another significant player in medical digitalization research is the University of Agronomic Sciences & Veterinary Medicine Bucharest, with 49 articles and 18 collaborations (e.g., Medicine & Pharmacy Cluj-Napoca, Romanian Academy of Science). It ranks second, as an active Romanian institution involved in both its own research and international networks. Similarly, Iuliu Hațieganu University of Medicine & Pharmacy Cluj-Napoca, with 46 articles and 31 collaborations, ranks third, standing out with an extensive network of researchers and significant activity in international projects, such as collaborations with the University of Zurich, University of Oxford, Goethe University Frankfurt, etc.

Kunming Medical University in China ranks fourth, with 41 articles and 13 collaborations, indicating a focus on local research, while also demonstrating a tendency to expand its international networks through collaborations with institutions such as the Chinese Academy of Sciences and Yunnan University. Following closely is Carol Davila University of Medicine & Pharmacy Bucharest, which ranks fifth with 39 articles and 28 collaborations and is one of the most prominent institutions in Romania, demonstrating notable international activity, including collaborations with Johns Hopkins University, the University of Lisbon, the University of Cape Town, and others.

The University of Zurich, with 37 articles and 32 collaborations (e.g., Charité University Medicine Berlin, University of Bern, Karolinska Institute), ranks sixth. The University of Bucharest, with 36 articles and 23 collaborations, ranks seventh, highlighting its importance in medical digitalization research in Europe, often collaborating with the University of Cape Town and the University of Agricultural Sciences and Veterinary Medicine. The Technical University of Munich, with 36 articles and 24 collaborations, ranks eighth and is an institution with a significant international impact in the field of digital technologies applied to medicine, frequently collaborating with the University of Basel, Otto von Guericke University, and other centers.

Hannover Medical School, with 34 articles and 29 collaborations, ranks ninth, demonstrating prolific international collaboration with institutions such as the Medical University of Vienna, the University of Zurich, and Babes-Bolyai University. Ludwig Maximilian University Munich, with 37 articles and 25 collaborations (e.g., the University of Cologne, the University of Birmingham, and Semmelweis University), completes the top ten institutions in this ranking.

In conclusion, the analysis of academic and research institutions involved in medical digitalization provides a detailed perspective on the centers of excellence that contribute significantly to the advancement of this field. The ranking answers RQ5 and highlights not only the number of publications and international collaborations but also the extensive networks of partnerships that support research in digital medicine. Institutions like Charité University Medicine Berlin and the University of Agronomic Sciences & Veterinary Medicine Bucharest play an essential role in technological development and strengthening international collaborations. Additionally, institutions in Romania, such as Iuliu Hațieganu University of Medicine & Pharmacy Cluj-Napoca and Carol Davila University of Medicine & Pharmacy Bucharest, stand out for their active involvement in relevant research and international partnerships, demonstrating that research in medical digitalization extends beyond the boundaries of major research powers to include smaller yet innovative centers. This diversity of institutions underscores the importance of international collaboration and partnerships in the field of medical digitalization for global scientific progress.

### 3.6. Co-Occurrence of Keywords and Emerging Trends

The analysis of keywords proposed by authors in the field of medical digitization plays a crucial role in understanding major research directions, current priorities, and the impact of digital technologies on the healthcare system. Identifying and evaluating the frequency of these concepts provides a clear perspective on how central themes in this field are addressed and interconnected within the specialized literature.

**Figure 7:**
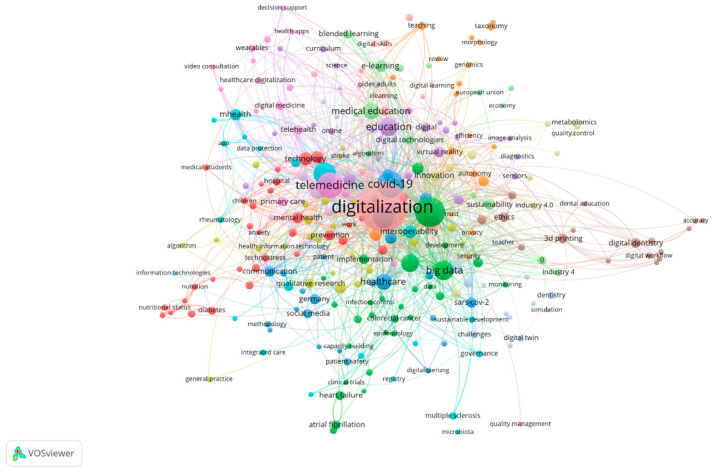
Co-occurrence of Keywords. Source: Author’s own processing using VosViewer.

The keywords identified in works on medical digitization reflect the essential themes and research directions that define this field. An analysis of their frequency reveals current priorities, contributing to the development of a more efficient, accessible, and sustainable healthcare system. This integrated perspective offers a framework for understanding how these concepts complement each other and influence the field of digital health. According to the results obtained from processing the Web of Science database, as presented in Figure 7, 11,778 keywords proposed by authors were identified. For clearer visualization of the most frequently used keywords, a threshold of a minimum of 10 occurrences was applied for a keyword to be included in the final map, resulting in 245 terms that meet this minimum condition.

First, “digitalization”/“digitalisation” (410/71 occurrences), the central term, highlights the fundamental transformation of healthcare systems through digital technologies. By integrating solutions such as electronic health records (EHR) and automating clinical processes, the number of errors is reduced and access to information is improved. This technological foundation is essential for supporting other innovations. Next, concepts such as “artificial intelligence” (139) and “machine learning” (51) are pillars of technological advancement, being used in diagnosis, imaging analysis, and treatment personalization. These technologies not only support the analysis of large data volumes (“big data”, 79) but also contribute to streamlining clinical decision-making, thereby accelerating medical discoveries.

Another significantly developed area is “telemedicine” (124), which became indispensable during the “COVID-19” pandemic (117). These solutions have demonstrated the ability of technology to extend access to healthcare, ensuring the continuity of treatments even in times of crisis. In this context, the concepts of “health” (117), “care” (96), and “management” (76) emphasize the main goal of digitization: improving the quality of patient care and optimizing resource management.

Moreover, the terms “technology” (108), “digital health” (78), and “ehealth” (60) highlight the importance of technological infrastructure in transforming healthcare systems. These technologies create new opportunities for providing integrated and efficient healthcare services, while concepts like “system” (62), “implementation” (55), and “model” (55) emphasize the need for well-planned strategies to maximize their impact.

On the other hand, terms such as “impact” (85) and “education” (81) reflect the digital effects on the training of medical staff and the improvement of access to information (“information”, 52). Education is a crucial element for helping healthcare professionals adapt to new technologies and for increasing patient trust in digital solutions.

Additionally, it is observed that terms identified, such as “digital transformation”, “innovation”, “healthcare”, “risk”, “internet”, “challenges”, “validation”, etc., although appearing with a frequency under 50, are significant in shaping current research trends and the connections between medicine and digitalization. These keywords highlight essential directions in this field. Thus, the term “digital transformation” reflects the integration of digital technologies into healthcare systems to improve services, efficiency, and accessibility, while “innovation” suggests the emergence of new technological solutions, such as artificial intelligence, telemedicine, and connected medical devices. “Healthcare” represents the central field benefiting from digitization, marking significant transformations in the delivery of medical services.

“Risk” refers to the risks associated with digitalization in medicine, such as data security, technological errors, and adoption barriers, while “challenges” highlights the obstacles to implementing digital solutions, including high costs, resistance to change, and the need for adequate regulations. “Internet” indicates the foundational digital infrastructure that enables the development of solutions such as e-health, online consultation platforms, and electronic records. Last but not least, “validation” underscores the need for clinical and technical validation of digital solutions before their widespread adoption.

These concepts are essential for understanding how digital technologies shape medical research and practice. They can serve as a foundation for analyzing the interconnections between fields and for identifying future research opportunities.

Consequently, the analysis of keywords demonstrates a complex interconnection of themes, highlighting the role of each concept in modernizing medical practices and providing an answer to RQ6. These priorities explain the significant number of co-citations among authors contributing to this field, highlighting the relevance of their research in shaping a patient-centered digital healthcare system.

### 3.7. Most Cited Papers in the Field

The analysis of the most cited papers is an essential step in understanding the impact and main research directions within a field. In the context of digitalization in medicine, highly cited papers indicate key contributions that have significantly influenced the development of theories, practices, and technologies in this area. These studies are crucial for identifying emerging trends, research priorities, and practical applications that have shaped digital transformations in medicine.

The need for such an analysis stems from several key considerations. First, highly cited papers reflect their relevance within the scientific community and clinical practices, helping to solidify theoretical foundations and develop innovative technological solutions. Secondly, these studies allow the identification of interdisciplinary fields successfully integrated into medicine, such as tissue engineering, artificial intelligence, and biological data analysis. Finally, by analyzing these papers, knowledge gaps and future research directions can be identified, thereby optimizing digitalization strategies in the medical field.

Therefore, the analysis of the most cited papers provides a comprehensive perspective on the impact and relevance of research in the field of digitalization in medicine, serving as a valuable tool for assessing the current state of knowledge and guiding future development directions. Ultimately, this approach not only strengthens scientific understanding but also improves technological applicability, directly impacting the quality of medical practice and patient outcomes.

**Figure 8:**
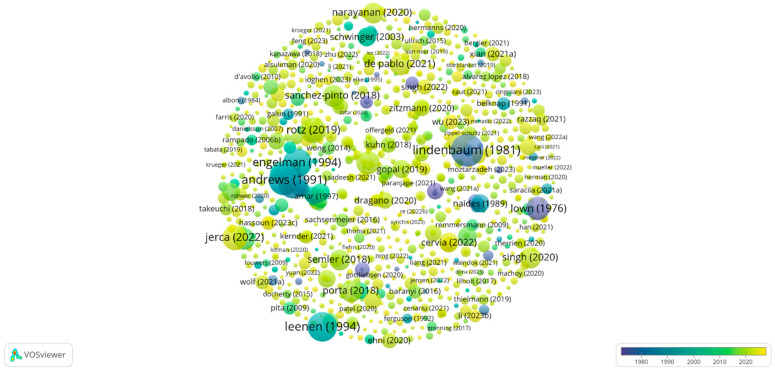
The most cited papers. Source: Author’s own processing using VosViewer.

Next, we analyzed the most cited papers from the total of 2606 papers according to the Web of Science database, establishing a threshold of at least five citations per paper. After this selection, we identified 996 relevant papers in the field of digitalization in medicine, each with at least five citations. Figure 8 illustrates this selection, offering an overview of the essential contributions in this field.

The most cited paper, with 359 citations, is “Inactivation of Digoxin by the Gut Flora: Reversal by Antibiotic Therapy”, authored by Lindenbaum et al. [70]. This research explores the conversion of digoxin into cardioinactive metabolites (DRPs) and the impact of gut flora on the efficacy of drug treatments. This contribution is essential for understanding how gut flora influences digital treatments and provides valuable insights into personalized medicine.

Second, with 319 citations, is the study by Andrews et al. [71], titled “Prevention of Supraventricular Arrhythmias After Coronary Artery Bypass Surgery”. This research contributes to the field of digitalization by proposing evidence-based therapeutic approaches that can be integrated into technological solutions for improving patient treatment and monitoring, thereby reflecting innovations in modern medicine.

The paper “Markers of Mouse Macrophage Development Detected by Monoclonal Antibodies”, authored by Leenen et al. [72], ranks third with 293 citations. In the context of digitalization, this research contributes to the development of tools for diagnosing and monitoring pathologies such as inflammation and autoimmune diseases. Furthermore, the study supports the advancement of medical imaging technologies and biological data analysis, which are critical for the application of digitalization in healthcare.

Another notable example is the paper “Fast-track Recovery of the Coronary Bypass Patient” by Engelman et al. [73], which ranks fourth with 248 citations. The study describes the implementation of a “fast-track recovery” protocol for coronary bypass patients, including digital monitoring methods such as preoperative education and early extubation. This contribution highlights how digital technologies optimize treatments and improve the efficiency of medical operations.

In fifth place, with 226 citations, is the research conducted by Falk et al. [74], titled “Digoxin for Converting Recent-onset Atrial Fibrillation to Sinus Rhythm: A Randomized, Double-blinded Trial”. The results show a limited impact of digoxin on converting to sinus rhythm, emphasizing the study’s contribution to optimizing therapeutic protocols and improving the understanding of the role of drug therapy in clinical digitalization.

A more recent study, “Advances and Opportunities in the Exciting World of Azobenzenes”, authored by Jerca et al. [75], ranks sixth with 226 citations. This research explores the synthesis of red light-sensitive azobenzenes and their applications in controlled drug delivery. This study opens new horizons for integrating advanced materials into digital medicine.

In seventh place is the paper “Basis for Recurring Ventricular Fibrillation in the Absence of Coronary Heart Disease and Its Management”, by Lown et al. [76] with 188 citations, highlighting the use of digital systems for monitoring and adjusting treatments in real-time. This contribution was crucial for integrating digital technologies into the management of complex conditions.

Next, the systematic review “Healthcare Professionals’ Competence in Digitalisation” authored by Konttila et al. [77] has received 183 citations and analyzes the digital competencies required in healthcare. The paper emphasizes the importance of digital skills and offers recommendations for the effective implementation of technologies in medical systems.

Another impactful study is “Probability of Transition to Psychosis in Individuals at Clinical High Risk: An Updated Meta-analysis”, authored by De Pablo et al. [78], which ranks ninth with 162 citations. The study highlights how continuous digital monitoring can support early diagnosis and preventive interventions.

Finally, the paper “In Situ Bioprinting—Bioprinting from Benchside to Bedside?” by Singh et al. [79], which has received 157 citations, explores advances in in situ bioprinting. This emerging technology integrates automation and digitalization to revolutionize medical treatments.

Analyzing the most cited papers in the field of digitalization in medicine, we observe that the top spots are dominated by older studies that, although not directly focused on digitalization, had a significant impact on medicine and medical research. These foundational papers laid the groundwork for the development of innovative technologies and approaches, serving as cornerstones of scientific progress in the field.

Through their remarkable contributions, these studies underscore the fact that digitalization in medicine cannot be fully understood without recognizing the critical influence of previous research. They illustrate the link between tradition and innovation, demonstrating that current advancements are built on solid foundations developed over decades.

As a result, the analysis of the most cited papers in the field of digitalization in medicine highlights their significant contributions to technological development and the improvement of medical practices, thereby answering RQ7. Each selected study, through its focused theme and impressive citation count, reflects its relevance in promoting medical innovations, whether it involves optimizing treatments, advanced diagnostics, personalizing interventions, or integrating digital technologies into healthcare.

However, we cannot overlook the importance and valuable contributions of other papers that, although not among the most cited, still play an essential role in advancing knowledge in this field. Each study, regardless of its citation count, plays a critical role in strengthening the scientific foundation and supporting efforts for the digital transformation of medicine. Therefore, a holistic approach and recognition of the diversity of contributions remain fundamental for a comprehensive and in-depth understanding of this interdisciplinary field.

## 4. Conclusions

Digitalization in medicine represents a significant transformation of healthcare systems and has a considerable impact on how data, treatments, and services are managed for patients. As digital technologies, such as artificial intelligence, telemedicine, and Big Data solutions, become increasingly integrated into practice, they not only enhance efficiency and accessibility but also pose new challenges related to regulations, information security, and the professional training of medical personnel. In this paper, we analyzed the evolution of research in this field, identifying key authors and institutions, as well as the main trends regarding the applicability and impact of these technologies on public health.

The results of the analysis highlight the significant impact of digitalization in medicine. Among the most important conclusions is the identification of prolific authors such as Tomuleasa C., Radic J., Li Z., and Pisla A., whose recent contributions highlight the growing interest in health digitalization in recent decades. Technological advances and the need to adapt healthcare systems play a crucial role in this trend. Additionally, the analysis of international collaborations and research networks shows a strong global network supported by organizations such as the WHO and the European Commission, which are actively involved in promoting research and innovative policies.

The geographic distribution of research shows that countries such as Germany, Romania, the USA, and China are leaders in this field due to their advanced digital infrastructure and available academic resources. However, even smaller countries like the Netherlands and Switzerland demonstrate that academic excellence and well-designed strategies can compensate for a lack of extensive technological resources. Moreover, initiatives from countries like Japan, Spain, and France highlight the global importance of digital health, with each country adjusting its strategies to address its unique challenges and needs.

Regarding academic institutions, Charité University Medicine Berlin and the University of Agronomic Sciences & Veterinary Medicine Bucharest stand out for their relevant research and extensive international collaborations. Universities in Romania, such as Iuliu Hațieganu University of Medicine & Pharmacy and Carol Davila University of Medicine & Pharmacy, have demonstrated a significant capacity to produce innovative research, thereby strengthening the global network of researchers in digital health.

Furthermore, the analysis of keywords revealed major trends in the field’s research, including terms like “digitalization”, “artificial intelligence”, and “telemedicine”, which reflect the fundamental transition in healthcare systems. Technologies such as “machine learning” and “big data” are essential for analyzing large datasets, improving decision-making processes, and accelerating medical advancements. At the same time, concepts like “impact”, “education”, and “risk” highlight the challenges associated with digitalization, including the need for ongoing medical staff training and the management of security risks.

The most cited papers indicate that foundational studies have had a major impact on the development of digitalization by demonstrating the link between traditional research and current technological innovations. These contributions emphasize the importance of a solid scientific foundation to support technological progress in medicine.

Thus, the main conclusion of this study is that health digitalization plays a crucial role in transforming healthcare systems and that international collaborations and the integration of advanced technologies are essential for the success of this process. In this context, a holistic and adaptable research approach is necessary to address global challenges and support a patient-centered healthcare system.

The involvement of this study in medical practice suggests the need for a robust framework for implementing digital technologies that is adaptable and accounts for the specifics of each healthcare system. In this way, professionals in the field can use these findings to optimize decision-making processes and facilitate the integration of digital solutions, thereby contributing to more efficient resource management and improved accessibility to healthcare services.

From the perspective of the literature, this study contributes by identifying trends and challenges in health digitalization, filling existing gaps, and providing a detailed overview of the contributions made by researchers and academic institutions. The results obtained can guide future research directions, especially regarding the implementation and assessment of the impact of digitalization on healthcare systems worldwide.

On the other hand, a limitation of our study is regional bias, which may affect the representativeness of the included publications. This bias may arise from the uneven distribution of research activity across regions, leading to the over- or under-representation of certain geographical areas in the database. Moreover, this issue has been highlighted by Asubiaro et al. [80], who reveal disparities in journal representation across regions, disciplines, and languages, underscoring the need for a more inclusive and equitable research ecosystem. Another limitation is the inclusion of non-English languages. Although non-English languages are widely used, their role in academic communication remains relatively underexplored. Analyses suggest that English has increasingly become dominant in fields such as the natural sciences, social sciences, arts, and humanities [81].

In conclusion, this study supports the continued development of interdisciplinary research in digital health and contributes to the creation of a solid theoretical framework that supports the implementation of digital technologies in healthcare.

## Data Availability

Data are contained within the article.
